# Immune modulatory effects of oncogenic KRAS in cancer

**DOI:** 10.1038/s41467-020-19288-6

**Published:** 2020-10-28

**Authors:** Shaima’a Hamarsheh, Olaf Groß, Tilman Brummer, Robert Zeiser

**Affiliations:** 1 Department of Medicine I, Medical Center - University of Freiburg, Faculty of Medicine, University of Freiburg, Freiburg, Germany; 2 Institute of Neuropathology, University Medical Center Freiburg, Faculty of Medicine, University of Freiburg, Freiburg, Germany; 3 Centre for Biological Signalling Studies (BIOSS) and Centre for Integrative Biological Signalling Studies (CIBSS), University of Freiburg, Freiburg, Germany; 4 Institute of Molecular Medicine and Cell Research (IMMZ), Faculty of Medicine, University of Freiburg, Freiburg, Germany; 5 German Cancer Consortium (DKTK) Partner Site Freiburg, German Cancer Research Center (DKFZ), Heidelberg, Germany; 6 Comprehensive Cancer Centre Freiburg (CCCF), University of Freiburg, Freiburg, Germany

**Keywords:** Tumour immunology, Immunosurveillance

## Abstract

Oncogenic *KRAS* mutations are the most frequent mutations in human cancer, but most difficult to target. While sustained proliferation caused by oncogenic *KRAS*-downstream signalling is a main driver of carcinogenesis, there is increasing evidence that it also mediates autocrine effects and crosstalk with the tumour microenvironment (TME). Here, we discuss recent reports connecting *KRAS* mutations with tumour-promoting inflammation and immune modulation caused by KRAS that leads to immune escape in the TME. We discuss the preclinical work on KRAS-induced inflammation and immune modulation in the context of currently ongoing clinical trials targeting cancer entities that carry *KRAS* mutations and strategies to overcome the oncogene-induced effects on the immune system.

## Abstract (web summary)

Oncogenic signalling has been historically associated with sustained cancer cell-intrinsic proliferation, however its role in promoting tumour immunoresistance has also become evident. Here, Hamarsheh and colleagues review and discuss the preclinical work on the immune modulatory effects of oncogenic KRAS and the potential clinical application.

## Introduction

The classical well-known concept is that oncogenic signalling results in tumour growth by enhancing proliferation while reducing apoptosis. Over the past decade, increasing evidence has shown that certain oncogenic mutations mediate crosstalk with the immune system via oncogenic signalling. Several oncogenes have been identified to be constitutively active in cancers. RAS oncogenes represent the earliest and most studied oncogenes. The role of RAS mutations as contributors to several human cancers has become evident and the underlying mechanisms and molecular regulators have been further elucidated. RAS proteins are small GTPases, which act as molecular binary switches, where they lead to the activation of various signalling pathways, such as mitogen-activated protein kinase (MAPK), phosphoinositide 3-kinase (PI3K) and RAL-GEF, promoting a variety of crucial cellular processes such as cell proliferation, differentiation and survival in response to extracellular stimuli^1^. RAS family members are encoded by the highly homologous genes *HRAS*, *NRAS*, *KRAS4A* and *KRAS4B* genes, and their activating mutations are found in ~25% of all human cancers (COSMIC database, version 91), which makes them the most widely prevalent and frequently mutated oncogene family. Most mutations affect the *KRAS* isoform (~86%), where the frequency and distribution vary depending on the cancer type. For instance, *KRAS* mutations are predominant in pancreatic ductal adenocarcinoma (PDAC, ~98%), colorectal cancer (CRC, ~52%) and lung adenocarcinoma (LAC, ~32%)^2^.

Inflammation and inflammatory responses play crucial roles during tumorigenesis and affect immune responses as well as the efficacy of treatment regimens. Infiltrating immune cells participate in a complex crosstalk with cancer cells mediated by molecular mechanisms within the tumour microenvironment (TME). The ability of cancer cells to evade immunological destruction but also tumour-promoting inflammation are both hallmarks of cancer^3,4^. Although the immune system is involved in the detection and destruction of tumour cells, immune cells can also act pro-tumorigenic^4,5^. The TME is comprised of innate immune cells, including macrophages, dendritic cells, neutrophils, natural killer (NK) cells and myeloid derived suppressor cells (MDSCs), T and B cells, in addition to stromal cells consisting of fibroblasts, adipocytes, endothelial cells and extracellular matrix (ECM)^6^. The different cell types within this complex and heterogeneous environment communicate, regulate and shape tumour growth through direct contact or via cytokine and chemokine production in an autocrine and paracrine manner^4^. The balance between pro- and anti-tumourigenic states is dictated by the expression of different immune mediators, modulators and the activation state of different cell types within the TME^4^.

The transforming function of oncogenic *RAS* mutations has been anticipated to be a result of their self-sufficiency in growth signals. However, the advancement in our understanding of carcinogenesis and its underlying mechanisms provided clear evidence that the effect of oncogenic *RAS* mutations extend beyond their sustained proliferation property. It has become more evident that oncogenic *KRAS* mutations mediate autocrine effects and crosstalk with the TME, particularly by promoting inflammation and evading the immune response and ultimately leading to tumour progression, invasion and progression^7,8^. In order to exert these effects, oncogenic KRAS expressed in tumour cells remodels the surrounding stroma cells by inducing several molecules such as cytokines, chemokines and growth factors. In addition, oncogenic KRAS co-operates with mutations of oncogenes or tumour-suppressor genes to induce a pro-inflammatory and/or an immunosuppressive stroma^9^. In this review, we discuss the crosstalk between oncogenic KRAS, inflammation and immune-modulatory mechanisms in cancer, with a focus on KRAS-induced NLRP3 inflammasome activation and programmed death-ligand-1 (PD-L1) expression. At last, we cover novel therapeutic approaches that target KRAS-induced inflammation and immune-modulatory mechanisms in cancer and review the agents currently being investigated in clinical trials.

## KRAS-induced inflammation

The relationship between inflammation and cancer goes back to the 18th century when Rudolf Virchow first hypothesised that cancer originates at sites of chronic inflammation, after observing the presence of leucocytes within neoplastic tissues^10^. Over the last two decades, the role of inflammation in tumorigenesis has been intensively studied and further clarified. The presence of several inflammation forms that differ by source of origin, mechanism of action, outcome and intensity has become more evident^11^. The association between inflammation and cancer can be viewed as two pathways, an extrinsic pathway triggered by infection-induced inflammatory signals and autoimmune diseases; and an intrinsic pathway caused by genetic alterations that promote inflammation and malignant transformation^12^. Regardless of the trigger, the stromal and immune cells within the TME communicate either by direct contact or via cytokines and chemokine production to control tumour growth. This crosstalk is regulated by the activation of different TME cell types and the expression of immune mediators and modulators, which, depending on the stage of tumour progression, tips the balance toward tumour-promoting inflammation or immune surveillance^4^.

**Fig. 1:**
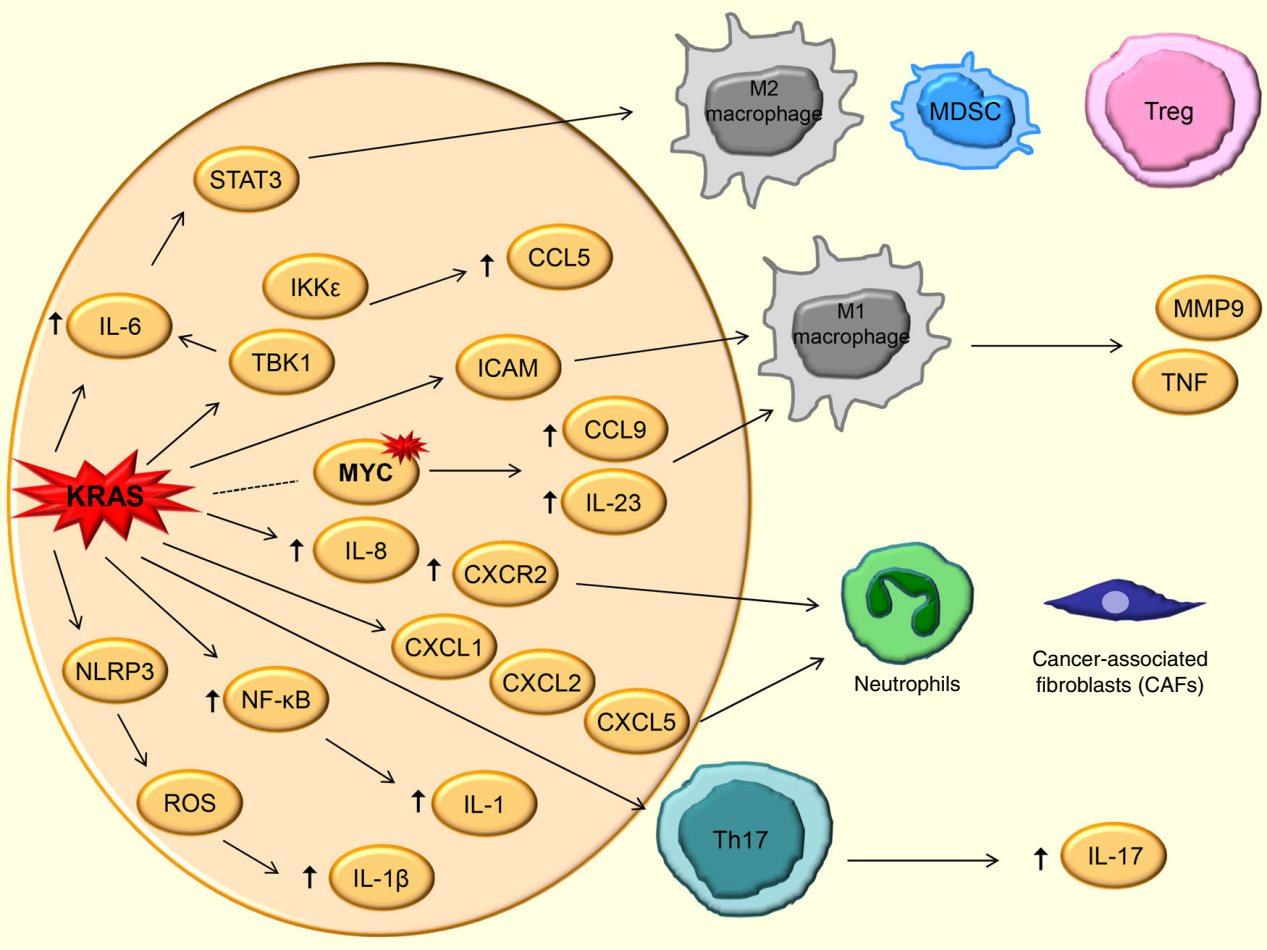
Pro-inflammatory effects of KRAS-induced inflammation in cancer. Pro-inflammatory effects mediated by the activation of transcription factors (STAT3), the production of cytokines (e.g., IL-6), the activation of NLRP3 inflammasome and the release of chemokines caused by oncogenic KRAS activation are listed. Representative immune cells in the tumour microenvironment (TME) affected by some of these signals are shown. The dotted line indicates co-occurring mutations.

*KRAS* mutations have been tightly linked to tumour-promoting inflammation and attributed to be a leading factor for carcinogenesis. This has been extensively studied and observed in the most common *KRAS*-mutated tumours arising from the epithelial linings of organs, particularly pancreas, colon and lungs. For instance, oncogenic KRAS induces several inflammatory cytokines, chemokines and signalling pathways that promote tumorigenesis and invasiveness in these cancers (Fig. 1)^13,14^. In addition, KRAS can promote stromal remodelling by inducing effects on endothelial cells, fibroblasts and ECM, which can also promote metastasis (reviewed by Dias Carvalho et al.^8^). Here, we review how KRAS induces several factors involved in inflammation-induced tumorigenesis.

Interleukin-6 (IL-6), a pleiotropic pro-inflammatory cytokine, has been proposed to be crucial for connecting inflammation and cancer^15,16^. Oncogenic RAS was shown to induce the secretion of IL-6 in different cell types, such as human kidney cells, fibroblasts, myoblasts and mammary epithelial cell, where this RAS-induced secretion of IL-6 is required for human tumour cell growth in vivo^16^. In addition, Brooks et al.^17^ demonstrated that IL-6 trans-signalling promotes KRAS-driven LAC. IL-6-mediated activation of Janus activated kinase 1 (JAK1) and the resulting downstream phosphorylation of the transcription factor signal transducer and activator of transcription 3 (STAT3) appears to be the main contributor to several tumorigenic cellular processes, particularly evident in lung and pancreatic cancer. For instance, oncogenic KRAS mutations have been shown to promote pancreatic intraepithelial neoplasias progression and PDAC, dependent on the secretion of IL-6 by myeloid cells and thus activation of STAT3 signalling pathway^18^. Corcoran et al.^19^ confirmed the critical roles of the IL-6/STAT3 axis during PDAC pathogenesis and found that phospho-STAT3 levels can serve as an effective biomarker for predicting response JAK2 inhibitors. The precise role of IL-6 in inflammation-driven pancreatic carcinogenesis and later diseases stages was not known. Later, Zhang et al. showed that IL-6 is required for the initiation of pancreatic cancer precursor lesions in the presence of inflammation. This effect was due to the synergism of oncogenic KRAS with IL-6 causing the activation of reactive oxygen species (ROS) via MAPK/ERK pathway^20^. In lung cancer, IL-6/STAT3 signalling induced by KRAS oncogene has contrasting roles in its initiation and progression^21^. The pharmacological inhibition of IL-6 in KRAS-mutant lung cancer causes the suppression of tumour progression, STAT3 activation and reduces frequency of tolerogenic macrophages, granulocytic MDSCs and regulatory T cells (T_reg_)^22^. In addition, researchers utilising a Kras^G12D^-driven lung cancer murine model with genetic deficiency for IL-6 reported that the presence of oncogenic KRAS mutation leads to a reduction in tumour growth and improved survival^23^. Although these studies provide evidence directed toward a pro-tumourigenic function of STAT3 signalling in KRAS-induced cancer, it has also been shown to act as a tumour-suppressor in LAC^24^. Through the STAT3 pathway, IL-22 which is secreted by CD4^+^ αβ and γδ T cells, stimulates oncogenic KRAS-driven lung cancer development by promoting a pro-inflammatory microenvironment^25^. These findings support the concept that in KRAS-driven cancer the IL-6/JAK/STAT3 axis promotes an inflammatory microenvironment and thereby enhances tumour progression.

Similar to IL-6, the CXCR2 ligand IL-8, also known as CXCL8, has been associated with inflammation, tumour growth and angiogenesis. For instance, IL-8 was identified as a transcriptional target of RAS signalling, which is required for the recruitment of endothelial cells and initiation of tumour-associated inflammation and angiogenesis^26^. The KRAS/IL-8 link via either MAPK or PI3K signalling pathways has been further supported by studies in human lung cancer cells lines and tumour specimens^27^ as well as human cell lines of colon^28^ and ovarian^29^ cancers. In addition, CXCR2 and two murine functional homologues of IL-8, KC and MIP-2, were investigated in a Kras^LA1^-induced murine lung cancer model and found to be highly expressed in premalignant alveolar lesions in addition to increased neutrophilic infiltration and a higher vascularity caused by vascular endothelial cells, respectively^30^. Lung tumorigenesis by inflammation was also shown to be mediated by IL-8/CXCR2 axis in addition to the recruitment of neutrophils and release of neutrophil elastase^31^. Recently, Awaji et al.^32^ have demonstrated that the KRAS/CXCR2 signalling in PDAC promotes phenotype alterations of cancer-associated fibroblasts (CAFs) to a more secretory function, inducing pro-tumourigenic cytokines, mediated by nuclear factor kappa-light-chain-enhancer of activated B cells (NF-κB) transcription factor. These reports show that IL-8/CXCR2 induced by oncogenic KRAS has a role in inflammation-induced tumorigenesis.

IL-1 and NF-κB are known as key mediators and inducers of inflammatory responses. A study performed by Ling and colleagues^33^, demonstrated that the activation of IL-1α and IKKβ/NF-κB by KRAS are required for the development of PDAC. The requirement for NF-κB has been also evident in lung cancer, which also presented NF-κB and IKKβ as potential therapeutic targets in KRAS-induced tumours^34–37^. In addition, IL-1 signalling and the transcription factor GATA2 seem to be required for KRAS-driven non-small cell lung cancer (NSCLC)^38^. The studies indicate a fundamental role of IL-1 and NF-κB in KRAS-induced inflammation in solid tumours.

Several chemokines have been implicated in inflammation-induced tumorigenesis^39^. Chemokine C-C motif ligand 5 (CCL5), also known as Regulated upon Activation, Normal T-cell Expressed and Secreted (RANTES), mediates migration and chemotaxis of cells and is expressed by several cell types such as fibroblasts, epithelial cells, tumour cells and immune cells. Although CCL5 is implicated in tumour progression and its elevated levels are detected in several cancers, it also promotes anti-tumour immunity via the recruitment of T cells and dendritic cells to the TME^40^. Research has uncovered a role for CCL5 in KRAS-induced lung cancer, where it is involved in a cytokine circuit along with IL-6 and STAT3 driving tumorigenesis^41^. This circuit appears to be mediated by IKK-related kinases TBK1 and IKKε, as the inhibition of JAK2/TBK1/IKKε leads to the disruption of this circuit and impairment of lung cancer growth^41^. In addition, RAS-expressing breast cancer cells induce CCL5 secretion through the interaction with mesenchymal stem cells, which then enhance the invasion and metastasis potential^42^. These studies shed the light on a relatively less studied modulator of inflammation-induced tumorigenesis, suggesting that therapeutic targeting of CCL5 might result in improved outcomes in KRAS-mutant cancers patients,

Activating KRAS mutations in pancreatic acinar cells initiate signalling, which leads to the chemoattraction of M1 macrophages, particularly through the expression of intercellular adhesion molecule-1 (ICAM-1)^43^. The attracted macrophages release matrix degrading enzymes including matrix metalloproteinase 9 (MMP9), as well as cytokines such as tumour necrosis factor (TNF), which drive acinar cell metaplasia. The depletion of macrophages or the use of ICAM-1 neutralising antibodies attenuated the progression of KRAS-induced lesions^43^. This suggests ICAM as an attractive therapeutic target, which could be combined with either KRAS targets, MEK inhibitors or immunotherapeutic agents.

IL-17-producing T helper (Th17) cells are a subset of helper T cells that produce the pro-inflammatory cytokine IL-17, which have been implicated in inflammation-induced tumourigenesis. KRAS mutations lead to the recruitment of Th17 cells and IL-17 production and are associated with tumourigenesis in several cancers. In lung cancer, oncogenic KRAS restrictedly expressed in lung epithelial cells causes an accumulation of Th17 cells in tumour tissues. The absence of IL-17 results in a reduction of tumour cell proliferation and angiogenesis, decreased expression of pro-inflammatory mediators and recruitment of myeloid cells^44^. Similarly, the genetic ablation of IL-17C in a Kras^G12D^ lung cancer mouse model causes a reduction of tumour proliferation in the presence of nontypeable Haemophilus influenzae (NTHi)-induced chronic obstructive pulmonary disease-like lung inflammation^45^. This is accompanied by a reduction of recruited inflammatory cells into the TME and expression of tumour-promoting cytokines such as IL-6 and an improved response to anti-programmed death receptor-1 (PD-1) treatment^45^. McAlllister and colleagues showed that human pancreatic cancer precursor lesions are infiltrated by Th17 cells and that the cancer cells overexpress IL-17 receptor (IL-17R). In addition, using pancreatic intraepithelial neoplasia (PanIN) KRAS mouse genetic models, the group observed increased numbers of Th17 and γδ T cells in the pancreatic pre-neoplastic microenvironment, which caused PanIN initiation and progression by the production of IL-17^46^. In CRC patients, elevated levels of IL-17 were associated with KRAS mutations in a stage-specific fashion^47^. These results provide evidence for the role of Th17 cells in inflammation-induced tumorigenesis in KRAS-mutant cancers.

One of the key players in inflammation is the inflammasome, which is a danger-sensing multimeric protein complex that is part of the innate immune response. Most inflammasome-nucleating cytoplasmic sensor proteins belong to the nucleotide-binding and oligomerization domain (NOD)-like receptor (NLR) family. In response to pathogen-associated molecular patterns (PAMPs), damage-associated molecular patterns (DAMPs) or homoeostasis-altering molecular processes (HAMPs), inflammasomes form and activate their component caspase-1, leading to proteolytic maturation and release of the inflammatory cytokines IL-1β and IL-18^48^ and a lytic form of programmed cell death known as pyroptosis. The most widely studied, but at the same time, most elusive inflammasome is formed by NLRP3. Although inflammasomes in general are linked to immune responses against invading pathogens, the function of NLRP3 inflammasome in human cancers remains a conflicting topic, where some reports indicate that it provides a protective, anti-tumorigenic effect in certain cancers, whereas others show evidence of pro-tumorigenic roles^49,50^. Notably, analysis of somatic mutations in human cancers has suggested NLRP3 as an oncogene^51^.

In the last two years, a couple of studies demonstrated a role of NLRP3 in immune suppression in cancer. For instance, a group of researchers found that NLRP3 promotes the expansion of immune-suppressive macrophages and IL-10 secretion in PDAC^52^. Another study showed that the inhibition of NLRP3 in macrophages within the TME leads to suppression of metastatic potential of melanoma cells^53^. Interestingly, a recent report has shown an immunoresistance mechanism to the immunotherapeutic PD-1 blockade, where CD8^+^ T-cell activation in response to the therapy induced a PD-L1/NLRP3 inflammasome signalling pathway. Mechanistically, the groups showed an association of the PD-L1/NLRP3 inflammasome pathway to the recruitment of MDSCs into the tumour tissue causing impairment of the anti-tumour response^54^. Nevertheless, there was no direct link between *KRAS* mutations and the NLRP3 inflammasome until we recently reported that oncogenic KRAS causes the activation of NLRP3 inflammasome, which has roles in the pathogenesis of KRAS-driven myeloproliferation^55^. Using genetic mouse models as well as patient samples, we observed that the NLRP3 inflammasome had a key role in the development of several features of KRAS-mutant myeloid leukaemia including cytopenia, splenomegaly and myeloproliferation. In addition, the pharmacological inhibition of either NLRP3 or IL-1R led to an improvement of the disease phenotypes caused by the *KRAS* mutation. These findings in mice were reproduced in human chronic myelomonocytic leukaemia (CMML), juvenile myelomonocytic leukaemia (JMML) and acute myeloid leukaemia (AML) harbouring *KRAS* mutations^55^. Altogether, several lines of evidence have emerged supporting the pro-tumourigenic role of NLRP3 inflammasome in cancer. We demonstrated KRAS-induced NLRP3 inflammasome activation in leukaemia. However, whether the NLRP3 inflammasome is also activated in KRAS-induced solid tumours such as pancreatic and lung cancers remains elusive, and requires further investigation.

Oncogenic KRAS also co-operates with other oncogenic mutations in the induction of immune modulation. The co-occurring mutations in *KRAS* and *MYC* drive tumorigenesis via programming inflammation in the TME. This co-activation in a mouse genetic lung cancer model leads to highly proliferative and invasive adenocarcinomas, characterised by a highly inflammatory, angiogenic and immune-suppressed stroma. This stromal reprogramming is driven by CCL9 and IL-23, where they also mediate immune-modulatory events, which will be discussed in the following section^56^.

Altogether, these different lines of evidence derived from multiple independent groups support the concept of the key role of KRAS-induced inflammation in carcinogenesis and provide essential insights on possible targeting approaches.

## KRAS-induced immune modulation

Most solid tumours harbour infiltrations of varied immune cells, including myeloid and lymphoid lineage cells. The TME often presents with infiltrating immune cells which have immunosuppressive properties such as T_regs_, MDSCs, tumour-associated macrophages (TAMs), neutrophils and mast cells. These infiltrating immune cells grant cancer cells immune escape mechanisms to diverge away from CD8^+^ T cells or NK cell/T-cell-mediated killing, enhancing the ability of cancer cells to acquire novel mutations, to evolve and to rapidly grow^6^.

**Fig. 2:**
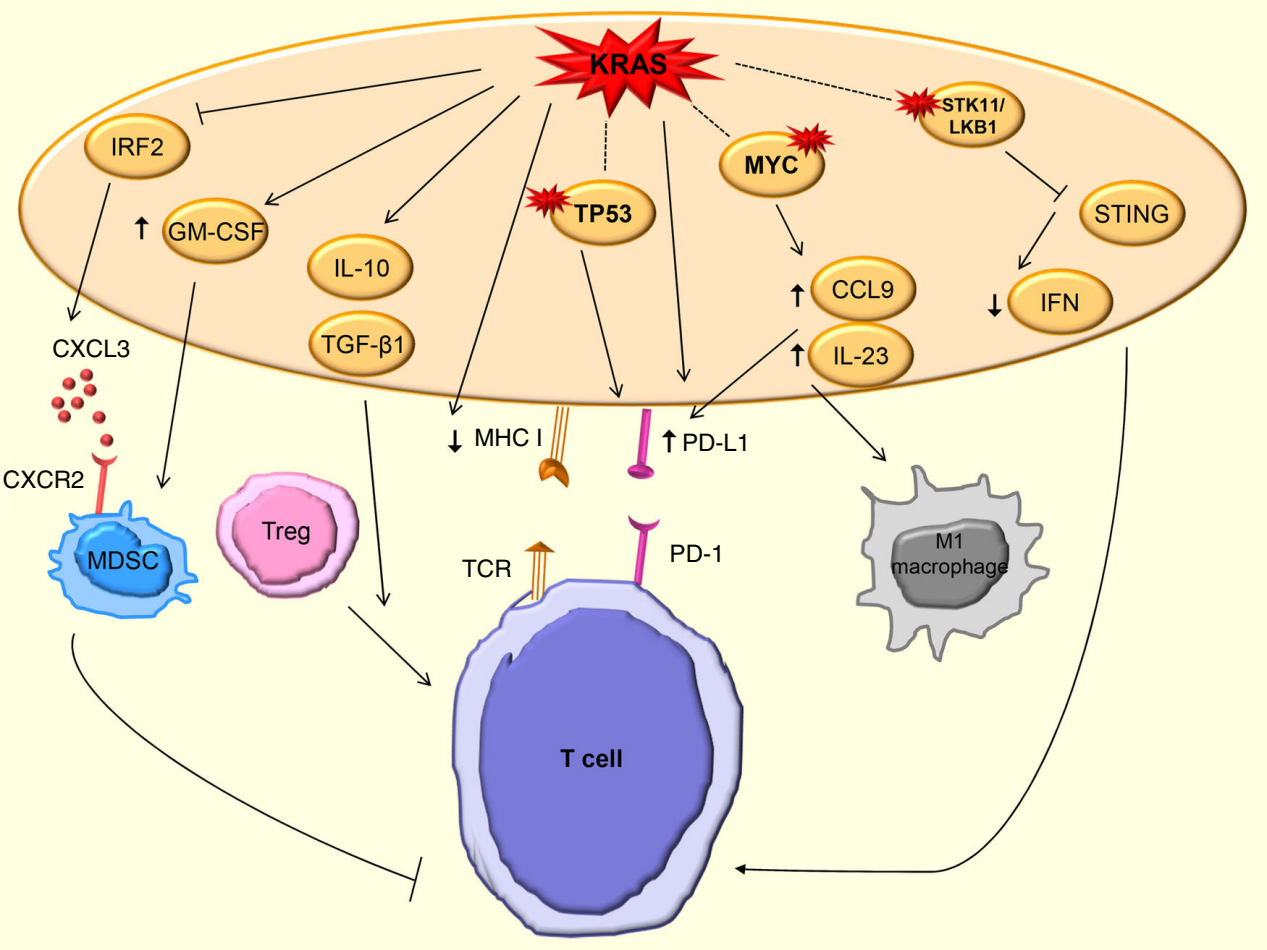
Effects of KRAS-induced immune modulation in cancer. Multiple intracellular downstream effects of KRAS on chemokine production (CXCL3), cytokine production, MHC expression and ligand expression are shown. Mutations are indicated by a red star symbol (activating mutations in MYC, inactivating mutations in TP53 and STK11/LKB1). The dotted line indicates co-occurring mutations.

KRAS-downstream pathways provide crucial roles in shaping the immune microenvironment, where the induction of NF-κB activates several cytokines and chemokines, including TNF-α, IL-1α/β, IL-6, CXCL1, 2, 5 and 8; and RAF/MAPK and PI3K also induce IL-10, transforming growth factor β (TGF-β) and granulocyte–macrophage colony-stimulating factor (GM-CSF) independent of NF-κB^57^. Here, we summarise the current knowledge of KRAS-induced immune-modulatory effects in cancer (Fig. 2).

In clinical research, a major goal is to overcome resistance to immunotherapeutic approaches targeting programmed death receptor-1 (PD-1) and PD-L1^58^. Several preclinical reports have shown evidence linking oncogenic *KRAS* mutations and PD-L1 expression in cancers, which reduces the tumour-specific T cells function in the TME. For instance, PD-L1 expression in KRAS-mutant lung cancer cell lines is regulated by MAPK-dependant transcriptional activity of AP-1 and partially by STAT3^59^. Another study demonstrated a direct association between *KRAS* mutations and the upregulation of PD-L1, mediated by ERK in human LAC cell lines and tissues^60^. Conversely, Lastwika and colleagues showed that the activation of KRAS-downstream pathway PI3K/AKT/mTOR in human LACs and squamous cell carcinomas, which tightly regulate PD-L1 expression both in vitro and in vivo. This was further supported by studies on patient samples, suggesting that oncogenic *KRAS* can cause immune escape by AKT/mTOR pathway via PD-L1^61^. Later, the mechanism causing *KRAS* to upregulate PD-L1 was shown to be through increases in PD-L1 mRNA stability via modulation of the AU-rich element-binding protein tristetraprolin (TTP), mediated by downstream MEK signalling^62^. The evaluation of patients with LAC showed a strong association between KRAS mutations and high PD-L1 expression^63^, which is contrasting to reports in CRC^64^. Canon and colleagues described the potential of combining the novel KRAS^G12C^ inhibitor AMG 510 with immune-checkpoint inhibitors, and showed that the combination of AMG 510 with anti-PD-1 blockade induced tumour cell killing, and improved the sensitivity of the TME to immunotherapy^65^. This study shed light on a great therapeutic potential of the simultaneous use of immunotherapy and compounds targeting oncogenic signalling pathways. More recently, Liu et al.^66^ demonstrated a correlation between KRAS mutations, increased PD-L1 expression and increased CD8^+^ tumour-infiltrating lymphocytes which associated them with an inflammatory TME and tumour immunogenicity. Overall, these results indicate an association between oncogenic KRAS activation and PD-L1/PD-1 expression and their immunosuppressive roles, which support therapeutic strategies to target KRAS-mutant cancers through abrogation of the microenvironment in pancreatic and lung cancer. Conversely, reports on CRC patients point towards the correlation of KRAS mutations and immunotherapy efficacy.

Another mechanism by which KRAS induces immunosuppression in cancer is via the induction of T_regs_ in the TME. Zdanov and colleagues showed that KRAS-mutant tumour cells induced the conversion of CD4^+^ cells to T_regs_. This suppression of T-cell activation was enhanced by the secretion of IL-10 and TGF-β1 through the activation of MEK/ERK/AP-1^67^. In lung cancer, the genetic ablation of T_regs_ in mutant KRAS transgenic mice developed fewer lung tumours, indicating that T_regs_ are required in lung tumourigenesis^68^.

KRAS mutations were also shown to be functionally involved in the downregulation of major histocompatibility complex (MHC) class I molecules, thereby causing the reduced ability of CD8^+^ cytotoxic T cells to recognise cancer cells^69^. In accordance, the knockdown of mutant KRAS by Smakman and colleagues in a poorly immunogenic CRC mouse cell line caused an improvement in immune response and tumour regression. This effect was attributed to the secretion of IL-18, an immune-stimulatory cytokine^70^. In addition, KRAS mutations were shown to upregulate GM-CSF in TME of pancreatic and CRCs, which enhances the infiltration of MDSCs causing an evasion of anti-tumour immunity^47,71,72^. This provides another role of oncogenic KRAS, where besides its direct pro-tumourigenic effect; it induces the recruitment of specific immune cells populations leading to immune escape.

Importantly, Liao et al. demonstrated the function of oncogenic KRAS in driving immunosuppression in CRC enabling tumour progression. KRAS appears to repress interferon regulatory factor 2 (IRF2), which results in high expression of CXCL3 that binds to CXCR2 on MDSCs, promoting their migration to TME. In addition, the authors showed that IRF2 overexpression overcame KRAS-induced resistance mechanisms to anti-PD-1 immunotherapy, and high IRF2 expression in CRC patients could be a predictive marker for anti-PD-1 therapy^73^. This report provides a novel role for IRF2 and presents it as a potential therapeutic target in KRAS-induced cancers.

Recently, Lowe and colleagues used immunocompetent mouse models of PDAC driven by *KRAS* and *Trp53* to investigate the efficacy of MEK inhibitor trametinib plus a cyclin-dependent kinase 4 (CDK4) and CDK6 inhibitor known as palbociclib in vivo. Their results indicated that this combination targets KRAS-directed oncogenic signalling, causing the suppression of PDAC proliferation through induction of retinoblastoma (RB) protein-mediated senescence. This combination therapy promoted tumour vascularisation through the induction of pro-angiogenic senescence-associated secretory phenotype (SASP), which led to enhanced drug delivery, endothelial cells activation and T cells infiltration, sensitising tumours to gemcitabine chemotherapy and PD-1 checkpoint blockade^74^. This study provides a clear rationale for the therapeutic potential of combining senescence-inducing therapies (i.e., trametinib and palbociclib) with chemotherapy or immunotherapy in PDAC and perhaps other cancers.

Apart from the pro-inflammatory function of KRAS, the oncogene often co-operates with other oncogenes or tumour-suppressor genes in the process of immune evasion in cancers. For instance, the co-activation of *KRAS* and *Myc* in lung cancer drives the recruitment of anti-inflammatory macrophages by CCL9 and IL-23 and exclusion of T, B cells and NK cells^56^. It was shown the *KRAS* and *TP53* collaborate in PDAC to promote tumour and immune invasion, by activating the ARF6/AMAP1 pathway, causing PD-L1 recycling and its cell surface expression^75^. Using genetically engineered murine models to study *TP53/KRAS*-driven lung cancer, researchers showed the effectiveness of combining MEK inhibitor with either anti-PD-1 or anti-PD-L1 in increasing anti-tumour immune responses and improving survival of lung cancer bearing mice. The response was mainly driven by increased tumour-infiltrating CD8^+^ and CD4^+^ T cells and a reduced percentage of MDSCs^76^. In addition, an integrated analysis using multiple-dimensional data sets containing genomic, transcriptomic, proteomic and clinical data of LAC patients showed that the co-mutated *TP53/KRAS* group exhibit increased intratumoural PD-L1 expression and high proportion of CD8^+^ T cells infiltrates. These observations were also consistent with the immunotherapeutic analysis which showed remarkable clinical benefits of patients with *TP53*, *KRAS* or *TP53/*KRAS-mutant cancer from PD-1 inhibitor treatment^77^. Besides, *STK11/LKB1* is one of the most commonly inactivated tumour suppressors in NSCLC, especially in those harbouring *KRAS* mutations. This co-mutation has been identified as a major driver of primary resistance to PD-1 blockade in KRAS-mutant LAC patients^78^. The loss of STK11/LKB1 causes suppression of stimulator of interferon genes (STING), a reduced expression of type I interferon genes and chemokines that promote T-cell recruitment, and tumour cells appear to lack PD-L1 expression, which altogether can be attributed to immunotherapy resistance^79^. Koyama et al.^80^ investigated the effect of this co-mutation in a KRAS-driven NSCLC mouse model and found that its inactivation led to aberrant cytokine production causing an increase in neutrophils, reduction in the numbers and function of tumour-infiltrating lymphocytes as well as the expression of PD-L1 in lung tumour cells. These reports suggest that co-occurring mutations with KRAS contribute to immune modulation in KRAS-driven cancers, mainly via increasing PD-L1 expression, which present as essential foundation towards further investigation in clinical trials.

These findings provide important insights into the immunosuppressive effects of oncogenic KRAS-mutations and they could lead to novel therapeutic concepts to reverse these phenotypes.

## Clinical translation

Despite over 30 years of research and extensive efforts to develop RAS-targeted therapies, most of the studies have failed to obtain clinically approved drugs or a one simple anti-RAS therapeutic approach and RAS has been often perceived as undruggable^2^. The approaches that have been used for targeting RAS include direct targeting, targeting of RAS modulators, targeting of upstream or downstream signalling or identification of RAS-specific synthetic lethality. Unfortunately, monotherapies targeted to RAS oncogenes have mostly failed following initial response owing to resistance occurring via different mechanisms such as feedback reactivation of RAS-downstream pathways. Nevertheless, substantial advances have been made lately, introducing promising compounds where some are undergoing clinical trials (reviewed in Moore et al.^81^). This includes approaches targeting KRAS^G12C^ oncoproteins or blocking GTP loading of KRAS oncoproteins, which indicate that direct targeting of KRAS might be achievable eventually, even though acquired resistance phenomena against these novel therapies might be inevitable^82^. Therefore, additional approaches that exploit oncogenic mechanisms operating in RAS transformed cells but also take the immunological consequences into account may help to identify vulnerabilities of the tumour, which could help to develop novel combination approaches that induce cure rather than palliation.

Several therapeutic approaches that target inflammation and immune modulation specifically in KRAS-mutant cancers have been undergoing investigation in clinical trials (Table 1), despite the fact that a larger number of trials have been studying these effects in different cancers often driven by KRAS mutations, regardless of the mutation status (Table 2).

**Table 1:** Clinical trials investigating therapeutic approaches targeting inflammation and immunomodulatory effects in KRAS-induced cancers.

Target(s)	Trial number	Phase	Drug(s)	Combination drug(s)	Cancer type	Current status
IL-6	NCT00841191	I/II	Siltuximab (CNTO 328)	—	Solid tumours	Completed
PD-1	NCT03777124	II	SHR-1210	Apatinib	NSCLC	Not yet recruiting
PD-1 or PD-L1	NCT04185883	I	PD-L1 or PD-1 inhibitors	Sotorasib (AMG 510)	Solid tumours	Recruiting
PD-1	NCT03785249	I/II	Pembrolizumab	MRTX849	Solid tumours	Recruiting
PD-1	NCT03299088	I	Pembrolizumab	Trametinib	NSCLC	Recruiting
PD-1	NCT03225664	I/II	Pembrolizumab	Trametinib	NSCLC	Recruiting
PD-1	NCT03991819	I	Pembrolizumab	Binimetinib	NSCLC	Active, not recruiting
PD-1	NCT03948763	I	Pembrolizumab	mRNA-5671/V941	NSCLC, CRC, PDAC	Recruiting
PD-1	NCT03004105	II	Durvalumab (MEDI4736)	Selumetinib	NSCLC	Withdrawn (insufficient funding)
PD-L1	NCT03637491	I/II	Avelumab	Binimetinib	Metastatic PDAC and KRAS-mutant solid tumours	Recruiting
JAK1/2	NCT02258607	I	Momelotinib	Trametinib	Metastatic NSCLC	Terminated

*CRC* colorectal cancer, *IL-6* interleukin-6, *JAK1/2* janus activated kinase 1/2, *NSCLC* non-small-cell lung cancer, *PD-1* programmed cell death protein 1, *PDAC* pancreatic ductal adenocarcinoma, *PD-L1* programmed death-ligand-1.

**Table 2:** Clinical trials investigating therapeutic approaches targeting inflammation and immunomodulatory effects in cancers often driven by KRAS mutations.

Target(s)	Trial number	Phase	Drug(s)	Combination drug(s)	Cancer type	Current status
IL-6 and PD-1	NCT04191421	I/II	Siltuximab (CNTO 328) and Spartalizumab (PDR001)	—	Metastatic pancreatic cancer	Recruiting
IL-8	NCT02536469	I	BMS-986253 (HuMax-IL-8)	—	Advanced malignant solid tumours	Completed
IL-8 and PD-1	NCT04123379	II	Anti-IL-8	Nivolumab	NSCLC and HCC	Recruiting
IL-1R	NCT00072111	I	Anakinra	—	Metastatic cancer	Completed
IL-1RAP	NCT03267316 (CANFOUR)	I/II	CAN04	—	Solid tumours	Recruiting
IL-1β	NCT03447769 - (CANOPY-A)	III	Canakinumab	—	NSCLC	Recruiting
IL-1β and/or PD-1	NCT03968419 - (CANOPY-N)	II	Canakinumab and/or pembrolizumab	—	NSCLC	Recruiting
IL-1β and/or PD-1	NCT03631199 - (CANOPY-1)	III	Canakinumab and/or pembrolizumab	Platinum-based doublet chemotherapy	NSCLC	Active, not recruiting
IL-1β	NCT03626545 - (CANOPY-2)	III	Canakinumab	Docetaxel	NSCLC	Active, not recruiting
IL-1β and PD-1	NCT03064854	I	Spartalizumab (PDR001) and canakinumab	Platinum-doublet chemotherapy	NSCLC	Active, not recruiting
PD-1 and IL-17 or IL-1β	NCT02900664	I	Spartalizumab (PDR001) and CJM112 or canakinumab	Trametinib	CRC, TNBC, NSCLC	Active, not recruiting
PD-1	NCT01295827 (KEYNOTE-001)	I	Pembrolizumab	—	Metastatic carcinoma, melanoma, or NSCLC	Completed
PD-1	NCT02130466	I/II	Pembrolizumab	Trametinib, dabrafenib	Advanced melanoma and solid tumours	Active, not recruiting
PD-L1	NCT02908672 (IMspire150)	III	Atezolizumab	Cobimetinib, vemurafenib	Metastatic or unresectable locally advanced melanoma	Active, not recruiting
ICAM and PD-1	NCT02043665	I	CVA21 (Coxsackievirus) and Pembrolizumab	—	NSCLC and bladder cancer	Completed
ICAM and PD-1	NCT02824965	I/II	CVA21 (Coxsackievirus) and pembrolizumab	—	NSCLC	Active, not recruiting
JAK1/2	NCT02244489	I	Momelotinib	Capecitabine and oxaliplatin	PDAC	Terminated

*CRC* colorectal cancer, *HCC* hepatocellular carcinoma, *ICAM* intercellular adhesion molecule, *IL-1RAP* interleukin-1 receptor accessory protein, *IL-1β* interleukin-1β, *IL-6* interleukin-6, *IL-8* interleukin-8, *JAK1/2* Janus activated kinase 1/2, *NSCLC* non-small-cell lung cancer, *PD-1* programmed cell death protein 1, *PDAC* pancreatic ductal adenocarcinoma, *PD-L1* programmed death-ligand-1, *TNBC* triple-negative breast cancer.

The anti-IL-6 monoclonal antibody, siltuximab, was tested as a monotherapy in KRAS-mutant solid tumours but showed limited clinical benefit (ClinicalTrials.gov identifier: NCT00841191)^83^. However, an ongoing phase I/II trial is currently investigating the combination of siltuximab and PD-1 inhibitor spartalizumab (PDR001) in metastatic pancreatic cancer (ClinicalTrials.gov identifier: NCT04191421). The forthcoming results will present important insights on the combination potential of anti-IL-6 and immunotherapy.

Although targeting IL-8 has not been investigated specifically in KRAS-mutant cancers yet, one study utilised the IL-8 inhibitor BMS-986253 as a single agent in advanced malignant solid tumours. The results showed well-tolerance in patients and confirmed decreases in serum IL-8 (ClinicalTrials.gov identifier: NCT02536469)^84^. This encouraged studying the potential of BMS-986253 in combination with the PD-1 inhibitor nivolumab in NSCLC and hepatocellular carcinoma (HCC) patients (ClinicalTrials.gov identifier: NCT03689699, NCT04123379). The future results would be important to explore the potential of IL-8 inhibitor combined with immunotherapy in KRAS-mutant cancers.

Owing to the fact that several immune-checkpoint inhibitors PD-1 or PD-L1 are effective in several cancer types, many clinical studies have become increasingly focusing on designing combinatory approaches with immunotherapy that could achieve synergistic effects. At present, the anti-PD-1 SHR-1210 is being investigated in combination with the vascular endothelial growth factor receptor-2 (VEGFR2) inhibitor, compared with chemotherapy drugs in KRAS-mutant stage IV NSCLC patients (ClinicalTrials.gov identifier: NCT03777124). Several companies such as Amgen and Mirati have already started exploring the combination of the promising KRAS-G12C targets Sotorasib (AMG 510) or MRTX849 with anti-PD-1 and anti-PD-L1 in patients with KRAS p.G12C mutant advanced solid tumours (ClinicalTrials.gov identifier: NCT04185883, NCT03785249). Notably, NSCLC patients with *TP53*, *KRAS* or co-occurring *TP53/KRAS* mutations showed favourable clinical benefit to anti-PD-1 treatment^77^. The PD-1 inhibitor, pembrolizumab, has been tested as a monotherapy NSCLC patients, where the results showed improved overall survival with a manageable safety profile (ClinicalTrials.gov identifier: NCT01295827)^85^. The combination of BRAF, MEK and PD-1 inhibitors in BRAF-driven melanomas, have favoured cancer cell death and displayed promising results in early clinical trials for metastatic melanoma, supporting the concept that inhibiting the oncogenic signalling may reduce immune evasion and promote response to immune-checkpoint inhibitor therapy (ClinicalTrials.gov identifier: NCT02130466)^86,87^. In KRAS-mutant NSCLC, pembrolizumab has been investigated in combination with standard chemotherapy, inhibitors of KRAS or its downstream effectors. For instance, three clinical trials are currently investigating pembrolizumab in combination with MEK inhibitors trametinib or binimetinib (ClinicalTrials.gov identifier: NCT03299088, NCT03225664, NCT03991819). Interestingly, the mRNA-derived vaccine targeting KRAS-mutant peptides (G12D, G12V, G13D and G12C), mRNA-5671/V941, is currently being tested in a combinatory regimen with pembrolizumab in KRAS-mutant NSCLC, PDAC and CRC patients (ClinicalTrials.gov identifier: NCT03948763). The forthcoming results will provide important insights on the safety and tolerability of these combinations in KRAS-mutant cancers.

One clinical trial is currently investigating the treatment of PD-L1 inhibitor avelumab along with MEK inhibitor binimetinib in patients with metastatic PDAC or KRAS-mutant solid tumours (ClinicalTrials.gov identifier: NCT03637491). Recently, the US Food and Drug Administration (FDA) approved the use of another PD-L1 inhibitor, atezolizumab, with MEK inhibitor cobimetinib and BRAF inhibitor vemurafenib for the treatment of patients with *BRAF*^V600^ mutation-positive advanced melanoma based on the results of the IMspire150 study (ClinicalTrials.gov identifier: NCT02908672). The trial results showed an increased progression-free survival and reduced relative risk for progression or death upon the addition of the atezolizumab to targeted therapy with vemurafenib and cobimetinib^88^. These results imply the potential synergy between immune-checkpoint inhibition and BRAF and MEK inhibitors.

As we have shown that KRAS causes NLRP3/IL-1β activation, targeting this axis may also be promising to achieve a therapeutic synergism with other anti-cancer therapies. Consistent with this concept, anti-inflammatory therapy with the IL-1β monoclonal antibody canakinumab in NSCLC, which is often driven by *KRAS* mutations, significantly reduced lung cancer incidence and mortality^89^. This finding motivated to further study the NLRP3/IL-1β axis in cancer. Ongoing studies in this field include a study in patients with metastatic cancer targeting interleukin-1 receptor (IL-1R) by anakinra (ClinicalTrials.gov identifier: NCT00072111) and a study targeting interleukin-1 receptor accessory protein (IL-1RAP) in patients with solid tumours (CANFOUR; ClinicalTrials.gov identifier: NCT03267316). In addition, a series of clinical studies initiated by Novartis (CANOPY) have been investigating the efficacy and safety of canakinumab in NSCLC as a monotherapy or in combination with chemotherapy and/or pembrolizumab (ClinicalTrials.gov identifier: NCT03447769, NCT03968419, NCT03631199, NCT03626545). Canakinumab is being investigated in combination with spartalizumab (PDR001) and platinum-doublet chemotherapy in NSCLC patients (ClinicalTrials.gov identifier: NCT03064854). Another trial is studying spartalizumab combined with either canakinumab, the anti-IL17A monoclonal antibody CJM112 or trametinib in NSCLC, CRC and triple-negative breast cancer (TNBC) (ClinicalTrials.gov identifier: NCT02900664). The forthcoming results are essential to build knowledge on the therapeutic potential of combined immunotherapy and IL-1β-targeted therapy and/or chemotherapy. Although these studies primarily target IL-1β, NLRP3 inflammasome activation also induces the production of mature IL-18, and therefore targeting NLRP3 directly may be more potent at interfering with the oncogene-induced immune modulation. Despite the development of potent and specific NLRP3 inhibitors, such as MCC950^90,91^, there are currently no clinical trials investigating their use in cancer patients.

On the other hand, different compounds that target specific pro-inflammatory cytokines, chemokines, NF-κB and JAK/STAT exist and some have been progressing to clinical studies. Ruxolitinib, which is a potent and selective JAK1/2 kinases inhibitor, is being tested in several clinical trials across different solid tumours. For example, the combination of ruxolitinib with capecitabine showed a trend of improved survival in patients with metastatic pancreatic cancer and evidence of systemic inflammation^92^. Another JAK1/2 inhibitor, momelotinib, also inhibits the IKK-related kinases TBK1 and IKKε. Although previous studies showed that pharmacological inhibition of TBK1 results in feedback ERK activation, a phase I clinical trial investigating the treatment of JAK1/JAK2 inhibitor momelotinib and trametinib in metastatic KRAS-mutant NSCLC patients who have failed chemotherapy provided no improved activity over single-agent trametinib at the doses used (ClinicalTrials.gov identifier: NCT02258607)^93^.

Another mechanism involved in KRAS-induced inflammation and tumorigenesis is regulated by ICAM. The inhibition of ICAM by CVA21 (Coxsackievirus) in combination with anti-PD-1 in NSCLC is currently being explored in two trials, and the initial results indicate that the treatment is well tolerated by the patients (ClinicalTrials.gov identifier: NCT02043665, NCT02824965). The investigation of this combination regimen further in other KRAS-induced cancers, particularly PDAC, would be beneficial.

In summary, investigating the potential of targeting KRAS signalling and its induced effects in cancer opens up new perspectives for combination approaches, whether with standard therapy, immunotherapy or targeted agents. Despite the limited number of clinical studies investigating drugs targeting inflammation and immunomodulatory effects specifically in KRAS-mutant cancers, the forthcoming results from the trials in cancers often driven by *KRAS* mutations will be provide crucial knowledge for future studies. Although it is early to evaluate the benefits of the mentioned approaches, the exploration of these opportunities could result in overcoming KRAS-resistance mechanisms and the forthcoming results are expected to bring new therapeutic benefit for patients with KRAS-mutant cancer.

## Future directions

Oncogenic *KRAS*-downstream signalling is a main driver of carcinogenesis. Here, we provide a perspective beyond the growth-promoting signals of oncogenic KRAS and discuss multiple lines of evidence for the concept that it impacts the immune microenvironment through a wide range of mechanisms including pro- and anti-inflammatory effects. Some alterations induced by oncogenic KRAS mutations in cancer promote tumour cell proliferation (mitogenic effects), whereas other immune-modulatory effects allow the tumours to evade immune-mediated attack by creating an immunosuppressive microenvironment.

Although our understanding of how KRAS-induced inflammation and immunomodulation contribute to tumorigenesis has widely improved over the past years, further studies are needed for a detailed understanding of the crosstalk between cells that harbour oncogenic KRAS and their microenvironment. In the past, most studies that analyse KRAS-mediated effects focus on pancreatic and lung cancers. We propose that it is important to investigate these mechanisms in other cancer entities where KRAS is less frequently mutated, such as myeloid leukaemia. A better understanding of the KRAS-induced effects and their therapeutic targeting could help to reduce resistance to immunotherapy. In future studies, it will be important to identify tumour-specific KRAS-induced mechanisms, in order to specifically identify and stratify patients that might benefit from suppression of tumour-promoting inflammation or immunotherapies for the eventual success of KRAS-targeted therapies. In addition, the characterisation of KRAS-downstream effector pathways will be essential for more-effective combination strategies.

In summary, targeting KRAS-induced effects on the immune system in cancer either solely or in combination with immunotherapies has great potential to increased clinical response in cancer patients.
